# Malignant Transformation in Vestibular Schwannoma: Clinical Study With Survival Analysis

**DOI:** 10.3389/fonc.2021.655260

**Published:** 2021-04-14

**Authors:** Jiuhong Li, Qiguang Wang, Menglan Zhang, Guisheng Zhang, Si Zhang, Xuhui Hui

**Affiliations:** ^1^ Department of Neurosurgery of West China Hospital, Sichuan University, Chengdu, China; ^2^ West China School of Medicine, West China Hospital, Sichuan University, Chengdu, China; ^3^ Department of Pathology of West China Hospital, Sichuan University, Chengdu, China

**Keywords:** vestibular schwannoma (acoustic neuroma), malignant, treatment, outcomes, prognostic factor

## Abstract

**Aim:**

Vestibular schwannomas (VSs) are generally considered benign tumors, and malignant transformation of VSs (MTVSs) are rare findings. The clinical features, treatment strategy, outcomes and prognostic factors remain unclear. We endeavored to analyze the natural history, management, outcomes and prognostic factors of MTVSs.

**Materials and Methods:**

The clinical features, radiologic findings, pathological investigations and surgical outcomes of 4 patients with MTVSs treated at the authors’ institution between 2010 and 2019 were retrospectively collected. Related literature published until December 2019 (63 articles, 67 patients) was evaluated. The authors also made a pooled analysis to evaluate the risk factors for overall survival (OS) time.

**Results:**

Of the 4 cases in our series, 3 cases were malignant transformation following previous treatment (surgery and radiosurgery) and 1 was primary MTVS. Of the 71 MTVSs from the literature, 27 were male and 39 were female, with the mean age of 47.2 ± 17.5 years old. Twelve patients (18.5%) were diagnosed with NF2 (15.4%) or NF1 (3.1%). Forty-three (61.4%) patients underwent previous treatment (surgery and/or radiotherapy) prior to the pathological diagnosis of MTVSs. The mean size of the MTVSs was 35.1 ± 13.2mm. The mean Ki-67 index was 30.6% ± 18.8%. Twenty-four (49.0%) patients underwent gross total resection, 25 (51.0%) patients underwent incomplete resection. Twenty-five (44.6%) underwent adjuvant radiotherapy (RT) postoperatively. During the average follow-up of 9.9 ± 9.5 months (range, 0-40 months), 37 (82.2%) patients developed a local recurrence or metastasis. Forty-seven (73.4%) patients died of tumor progression or postoperative complications. The overall 1-year and 2-year survival rate was 42.3% and 18.6% respectively. Log-rank testing for Kaplan-Meier survival analysis identified that size (P = 0.047) and adjuvant radiotherapy (P=0.001) were significant prognostic factors for OS. Multivariate analysis revealed that adjuvant RT was the only prognostic factor for longer OS (P = 0.005).

**Conclusions:**

MTVSs are rare, fatal disease, prone to recur and metastasize rapidly, resulting in death in most of the cases. We found that GTR did not improve the survival in MTVSs but postoperative adjuvant RT can significantly improve the OS, and we recommend early postoperative RT in MTVSs regardless of extent of resection.

## Introduction

Vestibular schwannoma (VS) is a common tumor originating from the nerve sheath of cranial nerve (CN) VIII, accounting for around 5%-6% of all intracranial tumors (1). Pathologically almost all VSs are benign tumors. Malignant change of vestibular schwannoma (MTVS), also called malignant peripheral nerve sheath tumor (MPNST) of the eighth CN, firstly reported by Norén et al. (2) in 1983, is very scarce, accounting for less than 1% of all vestibular schwannomas.

Because of its rarity, most previous studies of MTVSs have been case reports. MTVSs have an aggressive nature with a high recurrence rate, a tendency to metastasize, and are challenging to treat (3, 4). Most of these patients succumbed to this disease within one year after the diagnosis (5). Thus, it is vital to discuss the optimal treatment strategy and seek potential prognostic factors of MTVSs.

In the present study, therefore, we retrospectively analyzed the clinical features, radiologic manifestations, pathological findings and outcomes of 4 patients with MTVSs in our hospital. Additionally, we performed a systemic analysis of the reported cases of MTVSs to investigate the clinical features, optimal treatment strategy, and prognostic factors of this disease, which could be of potential interest to the neuro-oncology community. To the best of our knowledge, the present study is one of the largest case series and the first survival analysis on this topic.

## Materials and Methods

### Patient Population and Data Collection

From January 2010 to December 2019, 1329 cases of vestibular schwannomas underwent surgical resection in our institution, among which 4 patients were MTVSs. Patients accompanied with NF2 or NF1 disorder were included in our study. VSs patients without NF2 or NF1 disorder were termed sporadic VSs.

Patients met one of the following 4 sets of manifestations are diagnosed with NF2.

1. Bilateral vestibular schwannomas;
2. First-degree relative with NF2 plus unilateral vestibular schwannoma or two of meningioma, schwannoma, glioma, neurofibroma, posterior subcapsular lens opacity;
3. Unilateral vestibular schwannoma plus two of meningioma, schwannoma, glioma, neurofibroma, posterior subcapsular lens opacity;
4. Multiple meningiomas plus unilateral vestibular schwannoma or two of schwannoma, glioma, neurofibroma, posterior subcapsular lens opacity.

Two or more of the below criteria were required for NF1 diagnosis:

1. Six or more café au lait macules (>0·5 cm in children or > 1·5 cm in adults);
2. Two or more cutaneous or subcutaneous neurofibromas or one plexiform neurofibroma;
3. Axillary or groin freckling;
4. Optic pathway glioma;
5. Two or more Lisch nodules (iris hamartomas seen on slit lamp examination);
6. Bony dysplasia (sphenoid wing dysplasia, bowing of long bone +/– pseudoarthrosis);
7. First-degree relative with NF1.

We retrospectively reviewed the clinicopathological details, including patient age, sex, neurofibromatosis syndrome, radiological features, pathological findings, treatment history, treatment methods, and clinical outcome. An institutional review board exemption and a waiver of the requirement of the written informed consent were submitted and approved to perform this retrospective study.

All patients received surgery and the extent of resection (EOR) was determined by reviewing the postoperative MRI. Postoperative gd-enhanced MRI without signs of residual tumor is termed total resection (GTR); residual tumor smaller than 5×5×2mm is called near total resection (NTR); subtotal resection (STR) means that the residual tumor volume is less than 20% of the original tumor; partial resection (PR) means that the residual tumor volume is greater than 20% of the original tumor. Follow-up was performed at 3 months, 6 months, and then once a year after surgery. During the follow-up period, tumor metastasis and recurrence were monitored by enhanced MRI.

### Systematic Analysis

In addition, we collected the data of patients with pathologically confirmed MTVSs according to the PRISMA guidelines (6). PubMed was searched for case reports and series reported up to December 2019, using the following search strategy: *(Malignant peripheral nerve sheath tumor) OR (malignant[Title/Abstract]) AND (neuroma, acoustic[MeSH Terms]).* Also, the references cited in the selected reports were searched manually and reviewed to identify additional potentially eligible studies. Next, the clinical and radiological features including patient age, sex, NF disorder, tumor size, pathological subtype and MIB-1 (Ki-67) index were obtained; the treatment and outcome data were also extracted. The judgement of radiation-induced MTVSs was evaluated according to modified Cahan’s criteria (7, 8), which was outlined as follows:

1. benign tumor must be pathologically confirmed before irradiation;
2. a latency period is required between radiotherapy and tumor development (several months);
3. there must be a second tumor occurs within the radiation field;
4. the tumor must be histologically different from the primary tumor;
5. the patient should not have any genetic predisposition for cancer development.

### Statistical Analysis

Pearson’s chi-square test was used to compare categorical variables. Overall survival (OS) time was defined as the interval from the time of surgery to death. OS was analyzed using Kaplan-Meier estimates and the log-rank test for comparisons. P values <0.05 were considered statistically significant. All statistical analysis was performed with SPSS version 23 software (SPSS, Chicago, IL, USA).

## Results

### MTVSs in Our Hospital

**Figure 1:**
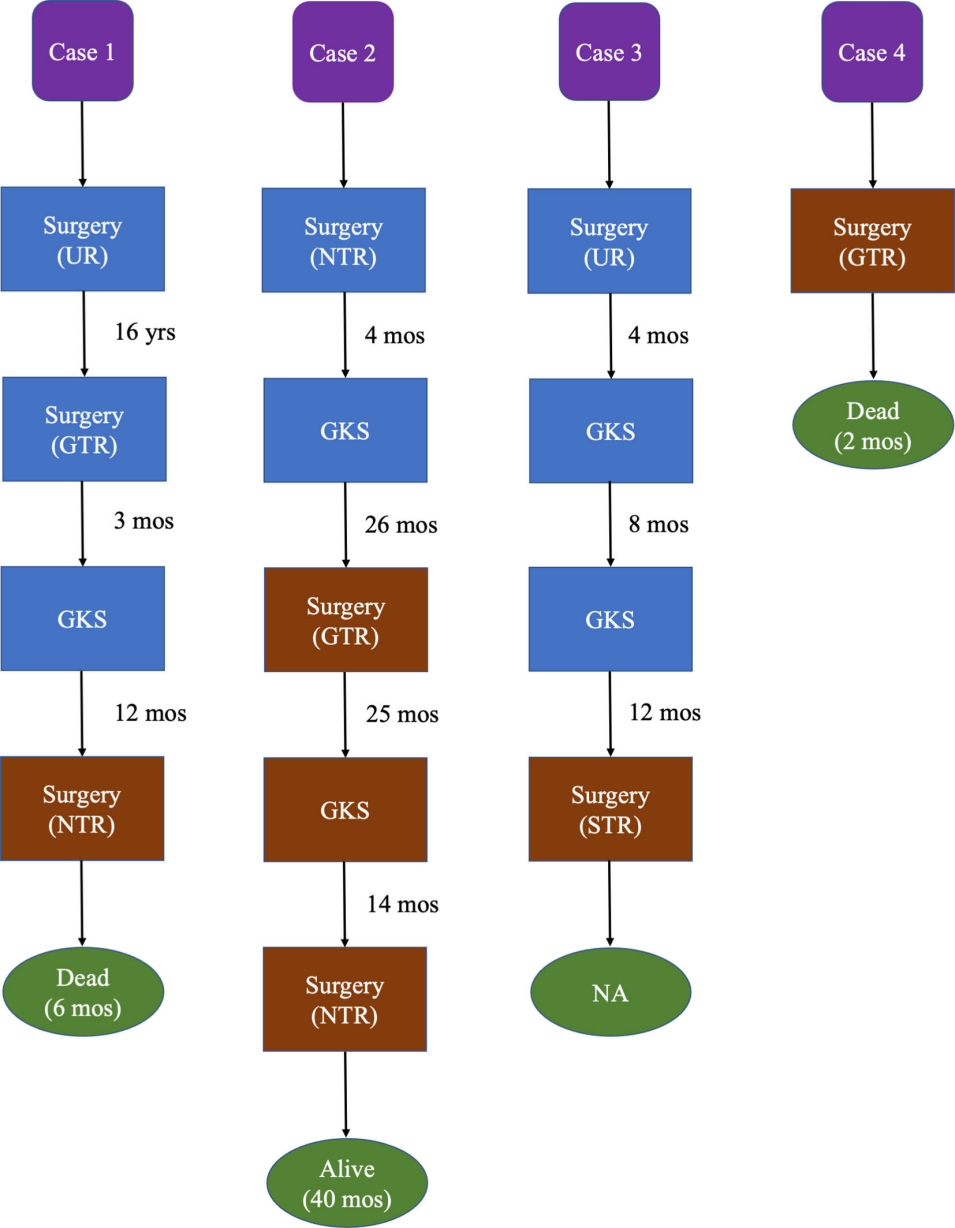
Treatment timeline and outcome of our 4 MTVSs. Brown blocks represent having pathological confirmation of MTVSs. Blue blocks represent no pathological confirmation of MTVSs. Green ovals represent the outcome and survival time since the pathological confirmation of MTVSs. GKS, gamma knife radiosurgery; GTR, gross total resection; mos, months; NA, not available; NTR, near total resection; STR, subtotal resection; UR, unknown resection; yrs, years.

The baseline characteristics of these 4 MTVSs were shown in **Table 1** (**Supplementary Materials**), and the treatment timeline and outcome were illustrated in **Figure 1**. Three of them had malignant transformation, which means they had previous operation(s) and the pathological diagnosis were all benign VS. The other one patient was primary MTVS without previous treatment. All four patients were female. The mean age at diagnosis was 51.0 ± 14.4 years old (range, 32-67). None of these patients had a history of NF2 or NF1. All 4 patients had facial nerve paralysis (House-brackmann grade III-IV) and hearing disorder. The mean size of the MTVSs was 37.5 ± 16.8 mm (range, 22-60). Of the four patients, one patient underwent two operations and gamma knife radiosurgery (GKS) previously, two patients received one operation and GKS, another one patient did not receive any kind of prior treatment. For the 3 patients received GKS prior to the pathological diagnosis of MPNST, the latency between radiation and pathological confirmation of malignant transformation was 12, 26 and 20 months respectively. As for the treatment, two patients underwent GTR, one patient underwent NTR and another one received STR. The pathological findings of our 4 MTVSs patients obtained in consecutive surgeries are summarized in **Table 2** (**Supplementary Materials**). The pathologic diagnosis was all MPNST, two patients with a WHO grade 3 (FNCLCC grade 2) (Case 1 and 2), among which one patient elevated to WHO grade 4 (FNCLCC grade 3) in recurrent MTVS (Case 2) and another two patients with a WHO grade 2 (FNCLCC grade 1) (Case 3 and 4). MIB-1 (Ki-67) index of MTVSs elevated compared with benign VSs collected at previous operations (30% vs. 2.0%&3.5%, 12.5% vs. 2.5% and 6.5% vs. 1.5% respectively). The mean MIB-1 (Ki-67) index was 17.9% ± 10.4% (range, 6.5-30.0%). After surgery, facial nerve function of House-brackmann (HB) level and hearing condition were the same as prior condition in all patients. One patient encountered aspiration and secondary pneumonia (Case 1) and 1 patient also encountered pneumonia (Case 2). During follow-up, two patients suffered from tumor recurrence. Metastasis was detected in none of these patients. One of them received the third operation due to tumor recurrence and was still alive at the time of last follow-up, with a survival time of 40 months. Two patients died 6 and 2 months after the diagnosis of MPNST respectively. One patient was lost to follow-up.

### Reported Data Review

All searches generated 238 reports, after carefully reading the titles, abstract, and the full-text; the last 63 articles were included. A total of 71 cases, including our case series, constitute the current body of data on MTVSs. The baseline data was demonstrated in **Table 3** (**Supplementary Materials**). Of the 71 cases, 27 were male and 39 were female, with the mean age of 47.2 ± 17.5 years old (range, 2–81). Twelve patients (18.5%) were diagnosed with NF2 (15.4%) or NF1 (3.1%). Out of the 71 cases, 8 (11.4%) patients underwent surgery prior to the pathological diagnosis of MTVSs, 16 (22.9%) patients underwent radiotherapy, 19 (27.1%) patients underwent both surgery and radiotherapy, while 27 (38.6%) patients received none previous treatment. The mean size of the MTVSs was 35.1 ± 13.2mm. The pathological diagnosis was MPNST in 49 (69.0%) patients, triton in 10 (14.1%) patients, sarcoma in 9 (12.7%) patients and melanotic in 3 (4.2%) patients. The mean MIB-1 (Ki-67) index was 30.6% ± 18.8% (range, 3.0-80.0%). Of 52 sporadic cases, 25 (48.1%) had a radiation history; of 12 NF patients, 5 (41.7%) had a radiation history. Of 10 NF2 patients, 6 had bilateral VSs, 2 had involvement of spinal cord and 3 had only a diagnosis of NF2 without detailed description. On MRI, these tumors depicted heterogeneous hypointensity on T1-weighted MRI and heterogeneous hyperintensity on T2-weighted MRI, and all tumors showed mild to moderate heterogeneous enhancement after administration of gadolinium. Twenty-four (49.0%) patients underwent GTR, 25 (51.0%) patients underwent incomplete resection (IR). Twenty-five (44.6%) underwent adjuvant radiotherapy (including RT 52%, GKS 40%, Others 20%) postoperatively.

**Figure 2:**
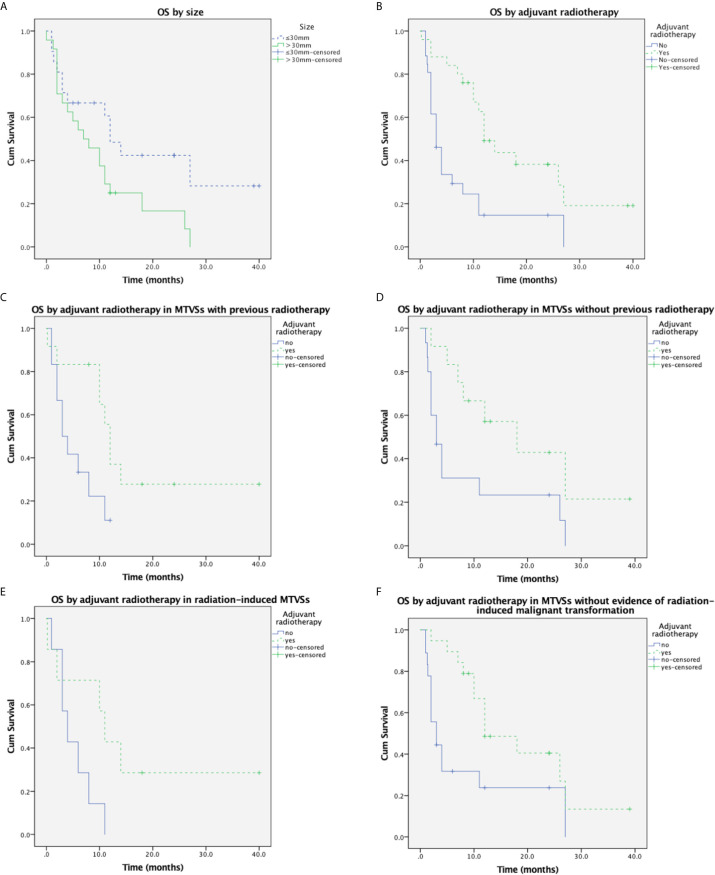
Cumulative overall survival in MTVSs. **(A)** Kaplan-Meier analysis of overall survival in 71 MTVSs patients based on size (≤30mm vs. >30mm). Tumor size>30mm was significantly associated with shorter OS time (p = 0.047). **(B)** Kaplan-Meier analysis of overall survival in 71 MTVSs patients based on postoperative adjuvant radiotherapy (yes vs. no). Patients underwent postoperative adjuvant radiotherapy was significantly associated with longer OS time (p = 0.001). **(C)** Kaplan-Meier analysis of overall survival in 35 MTVSs with previous radiotherapy revealed patients underwent postoperative adjuvant radiotherapy were significantly associated with longer OS time (p = 0.023). **(D)** Kaplan-Meier analysis in 35 MTVSs without a radiation treatment history revealed significantly longer OS in patients received postoperative adjuvant RT (P=0.024). **(E)** Kaplan-Meier analysis of overall survival in 20 radiation-induced MTVSs depicted postoperative adjuvant RT could not significantly improve OS time (p =0.073). **(F)** Kaplan-Meier analysis of the other 51 cases without evidence of radiation-induced MTVSs showed significantly longer OS in patients received postoperative adjuvant RT compared with those without postoperative adjuvant RT (P=0.012).

During the average follow-up of 9.9 ± 9.5 months (range, 0-40 months), 37 (82.2%) patients developed a local recurrence or metastasis. The recurrence rate was 78.9% (15/19) and 81.3% (13/16) in GTR and IR patients respectively. No malignant transformation of pre-existing tumors carried in NF2 MTVSs patients was reported. No *de novo* arising meningioma was reported in the radiation field in all MTVSs cases. Forty-seven (73.4%) patients died of the tumor progression or postoperative complications. The overall 1-year and 2-year survival rate was 42.3% and 18.6% respectively. Chi-square analysis showed that the incidence of NF (including NF2 and NF1) in patients with MTVSs is significantly higher compared to the incidence of NF (including NF2 and NF1) in patients with benign VSs (18.5% vs. 8.5%, P=0.007) (9). Log-rank testing for Kaplan-Meier survival analysis identified that size (P = 0.047) and postoperative adjuvant radiotherapy (P=0.001) were significant prognostic factors for OS (**Table 4**) (**Supplementary Materials**). Multivariate analysis showed postoperative adjuvant radiotherapy was the only prognostic factor for OS and patients with postoperative adjuvant radiotherapy had longer OS (P = 0.005, HR 0.359, 95% CI 0.175–0.737) (**Table 5**) (**Supplementary Materials**). Age (≤ 46 years or > 46 years), gender, MIB-1 index (≤ 30% or > 30%), previous treatment history (radiation and/or surgical history), EOR (GTR or IR) and pathologic subtype (MPNST or others) were not significant for OS. The Kaplan-Meier curves of size and postoperative adjuvant radiotherapy subgroup OS rates were demonstrated in **Figures 2A** and **B**. For the MTVSs patients with a radiation treatment history (35 cases) prior to the pathological diagnosis of MTVSs, log-rank testing for Kaplan-Meier analysis revealed significantly longer OS in patients received postoperative adjuvant RT (P=0.023) (**Figure 2C**); for the MTVSs patients without a radiation treatment history (35 cases) prior to the pathological diagnosis of MTVSs, Kaplan-Meier analysis also revealed significantly longer OS in patients received postoperative adjuvant RT (P=0.024) (**Figure 2D**). A total of 20 cases (including our 3 cases) totally conformed the modified Cahan’s criteria (7, 8) and their key characteristics are summarized in **Table 6** (**Supplementary Materials**) (10–21). For those 20 radiation-induced MTVSs cases, Kaplan-Meier analysis did not show significantly longer OS if the patients received postoperative adjuvant RT compared with those not received postoperative adjuvant RT (P=0.073) (**Figure 2E**); for the other 51 cases without evidence of radiation-induced MTVSs, Kaplan-Meier analysis showed significantly longer OS in patients received postoperative adjuvant RT compared with those without postoperative adjuvant RT (P=0.012) (**Figure 2F**).

### Illustrative Cases

#### Case 1

**Figure 3:**
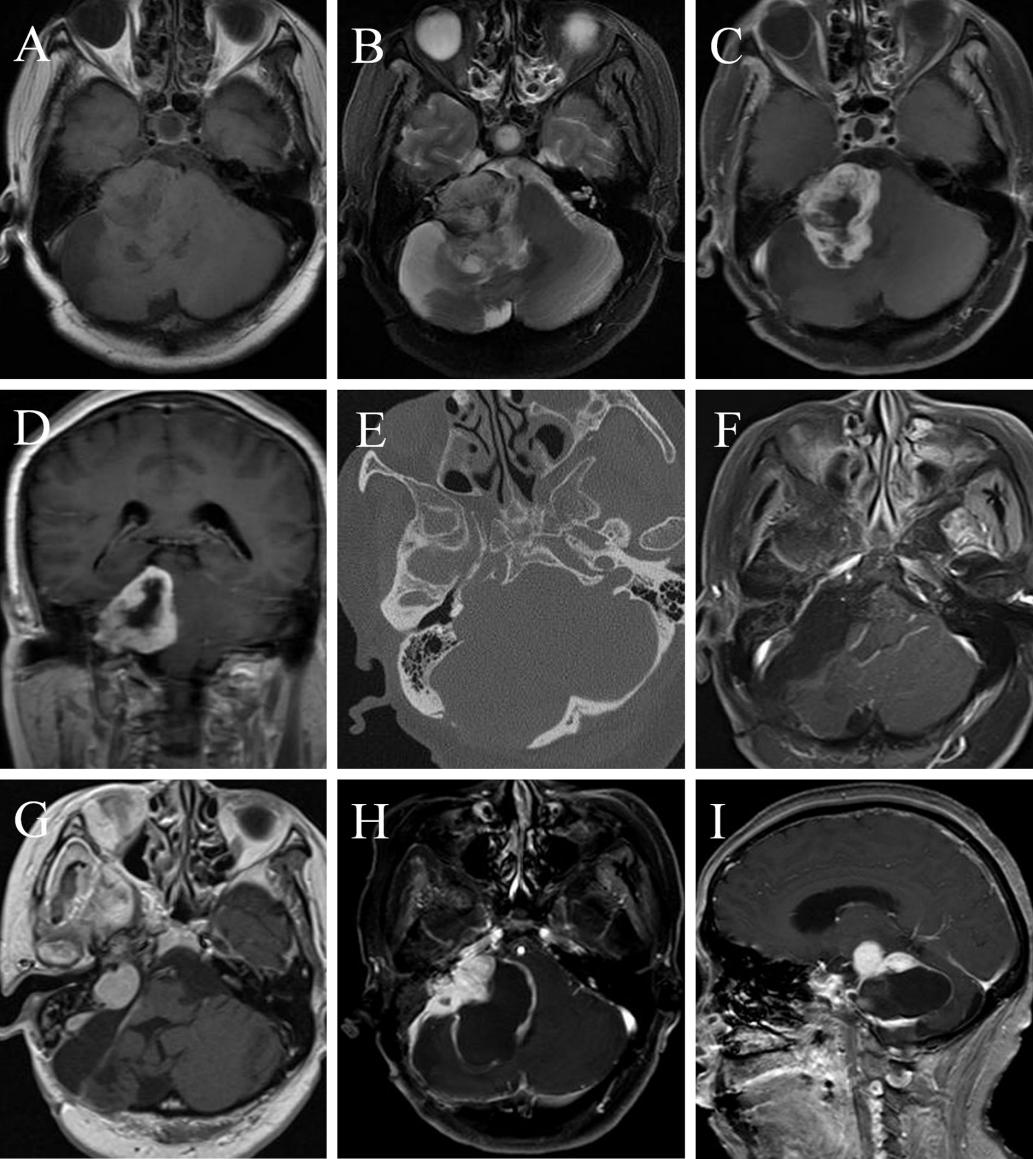
A MTVS with atypical radiological manifestation. Case 1. Magnetic resonance image (MRI) of a 51-year-old female showed a recurrent vestibular schwannoma, which was iso- to hypointense on T1-weighted **(A)** and heterogenous hyperintense on T2-weighted **(B)** images, with peritumoral edema. It was heterogeneously enhanced with cystic formation and irregular shape after administration of gadolinium **(C, D)**. A thin-slice CT scan of the internal auditory canal **(E)** showed the expansion of the right internal auditory canal and bone resorption destruction. The tumor was totally resected **(F)** and the histopathologic tests confirmed the diagnosis of a typical vestibular schwannoma. Gamma knife radiosurgery was prescribed to the patient 3 months later because of tumor recurrence **(G)**. One year later, contrast-enhanced MRI depicted a recurrent tumor with unusual manifestation-broad-based cystic-solid tumor with cyst wall enhancement, extending to petrous apex and ambient cistern **(H, I)**. She underwent the third operation in our department and the pathological diagnosis was MPNST (WHO grade 3/FNCLCC grade 2). Note: part of **Figure 3** has been demonstrated on Jiuhong Li’s Chinese Master’s Thesis.

**Figure 4:**
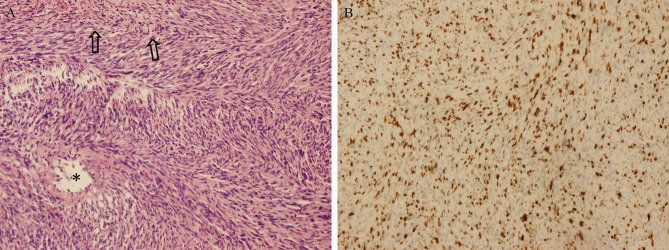
Pathological findings of MTVSs. Photomicrographs obtained during pathological examination of tumor tissue of Case 1. An H & E–stained section **(A)** showing the differentiation of MPNST with gland formation *(asterisks)* and necrosis *(arrows)*. Immunohistochemical staining for Ki-67 **(B)** was positive with 30%, revealing the diagnosis of MPNST (WHO grade 3/FCNLCC Grade 2). Original magnification ×200 **(A, B)**.

A 51-year-old female underwent a surgery of right vestibular schwannoma 16 years ago in another hospital. She presented with headache, facial paralysis for 3 months. Preoperative magnetic resonance image (MRI) revealed a recurrent vestibular schwannoma located at right cerebellopontine angle (CPA). The lesion was iso- to hypointense on T1-weighted and heterogenous hyperintense on T2-weighted images (**Figures 3A, 3B**). It was heterogeneously enhanced with cystic formation and irregular shape after administration of gadolinium (**Figures 3C, 3D**). A thin-slice CT depicted bone resorption destruction of the right internal auditory canal (**Figure 3E**). The tumor was totally resected (**Figure 3F**) and the histopathologic tests confirmed the diagnosis of a typical vestibular schwannoma. However, postoperative MRI 2 months later showed tumor recurrence with a maximum diameter of 21mm. Gamma knife radiosurgery (13Gy at 50% isodose line) was prescribed to the patient 3 months after the operation (**Figure 3G**). One year later, she presented with gait disturbance for 1 month and contrast-enhanced MRI depicted a giant recurrent broad-based cystic-solid tumor with cyst wall enhancement, extending to petrous apex and ambient cistern (**Figures 3H, 3I**), which manifested differently from typical VS. She underwent the third operation in our department and the pathological diagnosis was MPNST (WHO grade 3/FNCLCC grade 2), accompanied by partial epithelial differentiation (adenoid and neuroendocrine differentiation) (**Figure 4**). The patient aspirated while eating a liquid diet the first day after surgery and underwent tracheotomy due to lung infection. Six months later, the patient died of lung infection.

#### Case 2

**Figure 5:**
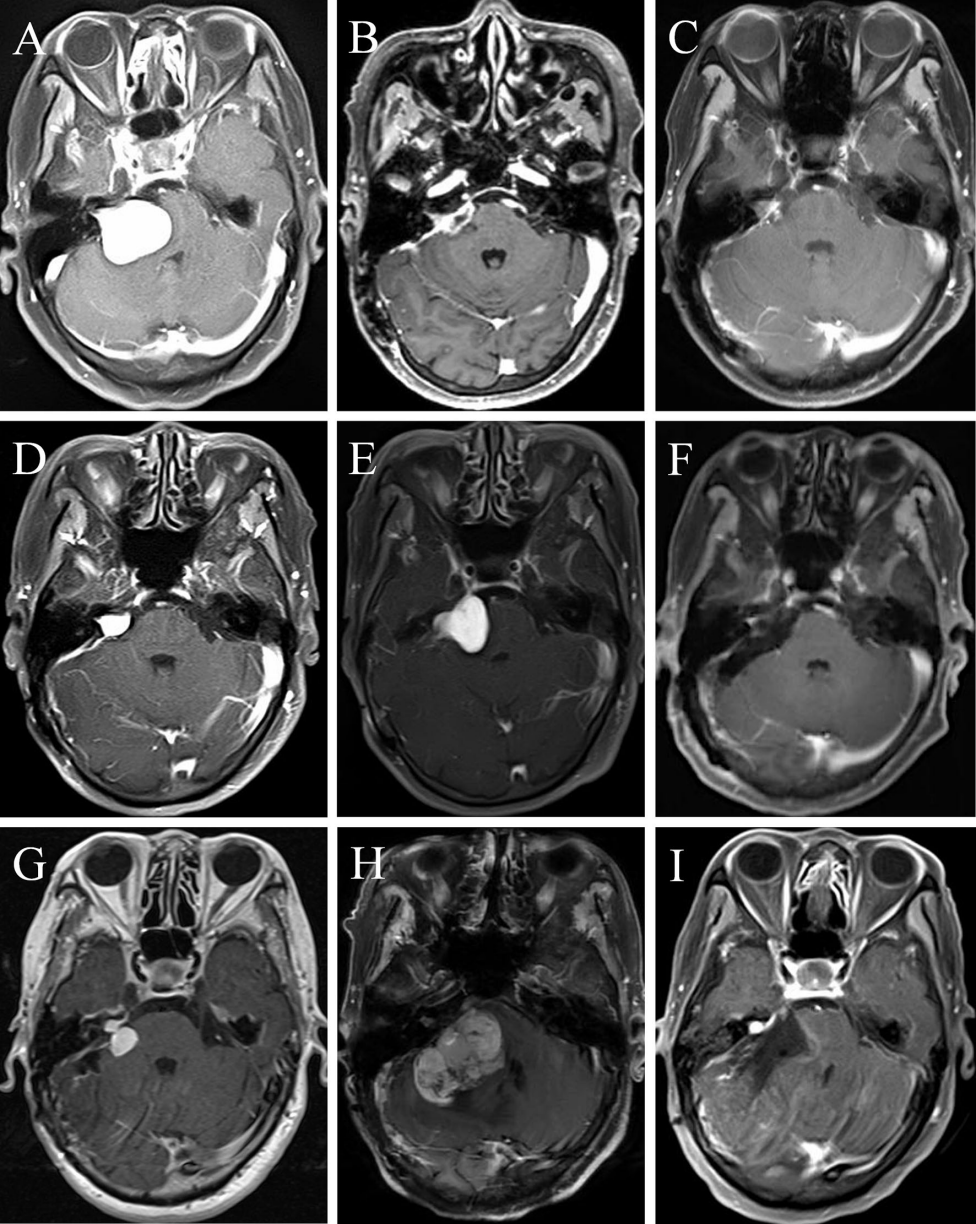
A female MTVS patient underwent multiple operations and radiotherapy with a 40 months’ survival time. Case 2. Preoperative contrast-enhanced magnetic resonance image (MRI) **(A)** of a 64-year-old female depicted a homogeneously enhanced tumor located at right cerebellopontine angle (CPA). After a NTR, enhanced MRI 3 months postoperatively **(B)** showed a remnant tumor and gamma knife radiosurgery (GKS) was conducted. Eight months after GKS, the tumor showed slight shrink on contrast-enhanced MRI **(C)**. However, enhanced MRI 2 years **(D)** and 2.5 years **(E)** after operation demonstrated regrowth of the tumor. She underwent the second operation and the pathological diagnosis was MPNST (WHO grade 3/FNCLCC grade 2). Postoperative MRI **(F)** confirmed gross total resection (GTR). Two years later, the tumor regrew again and GKS was prescribed **(G)**. One year later, the MPNST grew to a maximum size of 45mm with heterogenous enhancement **(H)**. The patient received the third operation and postoperative MRI **(I)** demonstrated tiny remnant tumor located at the roof of internal auditory meatus.

A 64-year-old female presented with right deafness for ten years and numbness of the right face and dysphagia for two months. Physical examination revealed gait disturbance. Preoperative contrast-enhanced MRI depicted a homogeneous enhanced tumor located at right CPA (**Figure 5A**). She underwent a surgery, and the EOR was NTR. Postoperative enhanced MRI 3 months later (**Figure 5B**) showed a remnant tumor and gamma knife radiosurgery (GKS) was conducted. Eight months after GKS, the tumor showed slight shrink on contrast-enhanced MRI (**Figure 5C**). However, enhanced MRI 2 years (**Figure 5D**) and 2.5 years (**Figure 5E**) after operation demonstrated regrowth of the tumor. The second operation was carried out and the pathological diagnosis was MPNST (WHO grade 3/FNCLCC grade 2). Postoperative MRI (**Figure 5F**) confirmed GTR. Two years later, the tumor regrew again and GKS was prescribed (**Figure 5G**). One year after that, the MPNST grew to a maximum size of 45mm with heterogenous enhancement (**Figure 5H**). The patient received the third operation, and the pathological diagnosis was MPNST (WHO grade 4/FNLCC grade 3), with moderate to severe atypia and high mitotic counts. Postoperatively, the patient had electrolyte imbalance, lung infection, and finally she recovered well with good facial nerve function (House-Brackmann grade II). Postoperative MRI (**Figure 5I**) demonstrated tiny remnant tumor located at the roof of internal auditory meatus.

## Discussion

Vestibular schwannomas are generally considered as benign tumors, and malignant changes in VSs are rare findings, which are not commonly known to the neuro-oncology community. At our center, MTVSs account for 0.3% of all VSs (4/1329). MPNST is a rare histologic subtype of sarcoma, mostly involving extremities and pelvis, and around half of them have NF1 syndrome (7, 8). Few cases of MPNST of the eighth nerve have been reported in the literature, and seldom large case series has been published as yet. MTVSs are aggressive malignant tumor with a tendency to recur or metastasize and poor clinical outcomes (5, 22), thus it is vital to detect the prognostic factors of MTVSs. In the current study, we have presented one of the largest case series of MTVSs and made a pooled analysis based on our case series and literature review.

### Clinical Features

According to the reported data review, MTVSs have a slight female predominance (1.4:1), which is different from benign VSs (~1:1) (1, 23). We found that most (54.7%) MTVSs were large (>30mm) tumors. It was notable that MTVSs seldom occur in pediatric patients, with only two (2 and 5 years old respectively) reported cases (24, 25). MTVSs had a high rate of NF disorder (including NF2 and NF1) (18.5%), which is significantly higher than that in benign VSs (8.5%) (9).

### Radiologic Investigation

MTVSs are difficult to detect preoperatively. After reviewing the literature, we found that most cases manifest as round/oval mass located in CPA, with heterogenous enhancement after administration of gadolinium, which share similar radiologic features with benign VSs. However, our findings also demonstrated that MTVSs could behave as typical malignant intracranial tumors, with serious damage to normal structures, such as broad-based manifestation and rim-enhancement (**Figures 3H, I**), massive and multifocal appearance (26, 27), extracranial involvement (28), diffuse infiltration of the parenchyma (4, 29, 30) and severe bone erosion (4). Thus, although rare, when encountering with atypical radiological features for a presumed VS, neurosurgeons should at least be reminded the possibility of MTVS.

### Pathologic Findings

Pathologically, MPNSTs are composed of conventional MPNST and divergent differentiated MPNST, for example, triton (rhabdomyoblastic differentiation), MPNST with glandular differentiation, sarcoma, angiosarcoma, osteosarcoma and malignant melanotic schwannoma (31). McMenamin et al. (32) reported 17 schwannomas with pathological confirmation of malignant transformation, including 4 cases of epithelioid malignant peripheral nerve sheath tumor and angiosarcoma, 9 cases of epithelioid malignant change. Based on pathological findings, MPNST could be classified as three grades according to French system (Fédération Nationale des Centres de Lutte Contre le Cancer or FNCLCC) soft tissue sarcomas. The system had a scoring standard based on tumor differentiation, mitotic count and tumor necrosis, classifying the MPNSTs according to the pathological findings and which may be useful for predicting the nature and outcome of MPNSTs (33, 34). For MTVS, MPNST FNCLCC grade 1, 2 and 3 is the same as WHO grade 2, 3, and 4 respectively, and benign VS belongs to WHO grade 1 tumor (31). However, the data of WHO grading or FNCLCC grading classification of the literature in MTVSs were hardly available and we advocate more investigations of detailed reports and description of WHO or FNCLCC grading classification of MTVSs.

Our case series showed that MIB-1 (Ki-67) index of MTVSs elevated greatly compared with their former benign VSs. Besides, MTVSs had a much higher mean MIB-1 (Ki-67) index (30.6% ± 18.8%) compared with benign VSs (<5%) according to our pooled analysis (35, 36).

### Treatment Strategy

For MTVSs, total resection should be considered as the optimal treatment. However, the majority of (61.4%) MTVSs had a treatment history such as surgery and/or radiotherapy. Thus, the tumor had a high rate of being adherent closely to crucial structures (eg. peripheral nerves and brainstem), which made complete resection challenging because of saving fundamental neurological function. Besides, for those MTVSs with diffuse infiltration, multifocal appearance, or severe bone destruction, it is less likely to achieve GTR.

According to our statistical analysis, postoperative adjuvant radiotherapy benefits the overall survival time in MTVS patients, which suggests that MTVS is sensitive in radiation treatment in a certain degree. Besides, postoperative adjuvant radiotherapy is as effective on OS in patients diagnosed with MTVSs after receiving radiation compared to patients who did not receive radiation before diagnosis. After reviewing the literature, we found that 20 cases out of 71 MTVSs cases (including our 3 cases) totally conform modified Cahan’s criteria (7, 8), which means the malignancy was most likely to be radiation-induced (**Table 6**) (**Supplementary Materials**). Our study revealed that postoperative RT could not significantly improve the OS in these radiation-induced MTVSs patients, which suggests radiation-induced MTVSs may be less sensitive to radiation treatment compared to MTVSs with spontaneous transformation. On the other hand, further studies with larger sample size of radiation-induced MTVSs are required to warrant a more persuasive conclusion regarding the role of postoperative RT in radiation-induced MTVSs. Our findings suggest that adjuvant radiotherapy may not cure MTVSs, but it is worth prescribing because it could prolong overall survival time, which were similar with Raper et al.’s study (37). They demonstrated a MPNST case and other 4 cases enrolled in the literature located at CPA, and most cases (4 out of 5 cases) had tumor shrinkage after stereotactic radiosurgery treatment. Thus they concluded that stereotactic radiosurgery may be considered to decrease the recurrence rate and progressive neurological decline. Overall, we recommend routine adjuvant radiotherapy as early as possible in MTVS regardless of the EOR.

As for chemotherapy, the evidence regarding the advantages of chemotherapy in the management of MTVSs is insufficient (38–43). Thus, we would not recommend the routine use of chemotherapy for MTVSs.

### Outcomes

According to our systemic analysis, it is no doubt that MTVSs had a poor prognosis, prone to (82.2% of all cases) recur with a rapid growth rate or metastasize to remote sites (spinal cord most likely) even when GTR was achieved. Most patients succumbed to the disease because of the progression of tumor or severe postoperative complications (eg. cerebral hemorrhage, infarction, pneumonia, hypotension and bulbar palsy) (44–48). The overall 1-year and 2-year survival rate was 42.3% and 18.6% respectively, which is much lower than spinal MPNST (87.5% and 50.0% respectively) (49). The following reasons could be attributed. First, the progression of the MTVSs result in increased intracranial pressure which could bring more catastrophic condition compared with spinal MPNSTs. Second, GTR is hard to achieve in MTVSs because of the preservation of peripheral fundamental structures, such as brainstem and peripheral cranial nerves. Finally, MTVSs had a higher rate of NF syndrome (commonly NF2) compared with spinal MPNSTs (commonly NF1) (18.5% vs. 14.0%), and NF2 patients with a malignant transformation of a vestibular schwannoma may have a poorer prognosis compared to NF1 patients with spinal MPNST (50). The longest survival time of MTVS patient was Case 2, who had a survival time of 40 months at the time of writing this paper.

### Prognostic Factors

According to our statistical analysis, size and adjuvant radiotherapy were significant prognostic factors for OS. And adjuvant radiotherapy was the only significant independent prognostic factor. A larger MTVS may make GTR harder to achieve, also it may have severer infiltration of surrounding structures which could lead to poor prognosis. Besides, our study did not find there is a significant different OS if MTVSs patients had a radiation treatment history. Carlson et al. (51) made a pooled analysis on 24 MTVSs cases without prior irradiation, and concluded that GTR was the only prognostic factor for improved disease-specific survival. However, according to our findings, regarding the EOR, Kaplan-Meier analysis failed to show any significant differences between the EOR and OS. This may suggest the aggressive nature of progression or recurrence even when tumor cells of MTVSs exist which is unrecognizable to the naked eye, which is quite different from benign VSs. On the other hand, the small sample size and unrecognized IR in patients thought to have undergone GTR may also be potential reasons. The reasons for the discrepancy between our study and Carlson’s regarding the prognostic role of EOR may be that our case series include the MTVSs with prior irradiation, which may include radiation-resistant cases, having a more aggressive nature even receiving GTR compared with those MTVSs without irradiation. In our study, 25 (44.6%) underwent postoperative adjuvant RT compared with 7 cases in the study carried by Carlson et al. (51), and the larger sample size may help us find the prognostic role of RT. As reported, postoperative progression, both recurrence and metastasis, can occur rapidly. Thus we recommend that in order to monitor tumor progression, strict and regular follow-ups of the entire central nervous system should be conducted at a shorter interval (every 3-6 months) compared with benign VSs.

### Possible Mechanism

The mechanism of malignant transformation of VSs remains unclear. We hold that spontaneous malignant transformation could occur in VS because 38.6% of MTVSs cases had no previous treatment prior to the pathological diagnosis of MTVSs (51).

The role of radiotherapy in the transformation of MPNSTs had been widely discussed in the literature. Some researchers believed that RT could increase the incidence of malignancy in VSs patients. Seferis et al. (8) suggested that radiation treatment could increase the incidence of malignant transformation to 10 folds in non-NF cases, but the risk is still extremely low (15-26 per 100000). However, some investigators believed that radiation may not contribute to malignancy because many cases had a progression phenomenon initially when radiotherapy prescribed which may represent malignant transformation had occurred prior to radiation exposure (30). A long-term follow-up study of nearly 5,000 cases (including 856 VSs) in Sheffield Hospital in the United Kingdom underwent radiotherapy found that any form of radiotherapy will not increase the risk of tumor malignant transformation (52). According to our institutional data, the incidence of radiation-induced MTVS is around 0.3%. Overall, we hold that radiotherapy could increase the incidence of malignant transformation in VSs, however, it is still a safe and effective way in tumor control in VSs and MTVSs.

Some authors believed that patients with VSs accompanied with NF2 have a higher risk of malignant transformation both spontaneously or after irradiation treatment compared with sporadic VSs (53–55). Conversely, King et al. (56) demonstrated that unirradiated NF2 patients do not have a higher rate of malignant transformation as they reviewed 1253 NF2 patients, and no spontaneous malignant transformation was found in unirradiated cases. Our study showed that MTVSs had a significantly higher rate of NF (including NF2 and NF1) disorder than that in benign VSs (18.5% vs. 8.5%). Notably, the proportion of radiation exposure (a radiation treatment history) of MTVS patients with NF disorder (including NF2 and NF1) was lower than that in sporadic MTVS patients (41.7% vs. 48.1%), which suggested that VSs patients with NF disorder (including NF2 and NF1) may have a higher rate of spontaneous malignant transformation compared with sporadic VSs. On the other hand, doctors may be less likely to prescribe RT treatment in VSs patients accompanied with NF2 or NF1 disorder because of possibly higher risk of malignant transformation compared with sporadic VSs patients may also be the potential reason. In summary, more studies focusing on genetic analysis as well as animal experiments, are necessary to elucidate the preferred mechanisms of malignant transformation in VSs.

### Study Limitations

The primary limitation of this study was its retrospective nature. Besides, because of financial limitations and local medical insurance policy restrictions, we could not obtain sufficient data regarding genetic conditions. Moreover, some were lost to follow-up. The small sample size makes it difficult to generate a persuasive conclusion. Hence, in order to get more valuable conclusion and maximize the sample size, we included patients from the literature and some intrinsic limitations should attention. Therefore, further investigations, especially large sample-size multicenter prospective studies on this rare subset of tumors are expected.

## Conclusions

MTVSs are rare, prone to recur and metastasize rapidly, resulting in death in most of the cases. Some MTVSs have distinguishable radiological features, such as broad-based manifestation and rim-enhancement, massive and multifocal appearance, extracranial involvement, diffuse infiltration of the parenchyma and severe bone erosion compared with benign VSs. We found that GTR did not improve the survival in MTVSs but postoperative adjuvant RT can significantly improve the OS, and we recommend early postoperative RT in MTVSs regardless of extent of resection. Strict and regular follow-ups of the entire neuraxis should be conducted at a shorter interval.

## Data Availability Statement

The original contributions presented in the study are included in the article/**Supplementary Material**. Further inquiries can be directed to the corresponding author.

## Ethics Statement

The studies involving human participants were reviewed and approved by Ethics Committee of West China Hospital of Sichuan University. The patients/participants provided their written informed consent to participate in this study.

## Author Contributions

JL and XH conceived the study design. QW, MZ, and GZ were responsible for patient recruitment and literature review. JL, QW, GZ, and SZ were responsible for statistical analysis. JL, GZ, and SZ drafted the article. All authors contributed to the article and approved the submitted version.

## Funding

This work was financially supported by Sichuan Province Science and Technology Support Program (No. 2017SZ0195) and the Youth Program of National Natural Science Foundation of China (No. 81801178).

## Conflict of Interest

The authors declare that the research was conducted in the absence of any commercial or financial relationships that could be construed as a potential conflict of interest.

## Abbreviations

CN, cranial nerve; CPA, cerebellopontine angle; EOR, extent of resection; GKS, gamma knife radiosurgery; GTR, gross total resection; IHC, immunohistochemical staining; IR, incomplete resection; MPNST, malignant peripheral nerve sheath tumor; MRI, magnetic resonance image; MTVS, malignant transformation of vestibular schwannoma; NF, neurofibromatosis; NTR, near total resection; OS, overall survival; PR, partial resection; RT, radiotherapy; STR, subtotal resection; UR, unknown resection; VS, vestibular schwannoma.
