# Supplementary material for: Malignant Transformation in Vestibular Schwannoma: Clinical Study With Survival Analysis
Source: Front Oncol. 2021 Apr 14;11:655260. doi: 10.3389/fonc.2021.655260 (PMC8079768; doi:10.3389/fonc.2021.655260)
Supplement: Supplementary file 1 [file DataSheet_1.zip › Supplementary Table 1.DOCX]

Supplementary Table 1: Baseline characteristics of our case series

| Pt. No. | Age (years),  Sex | NF | Symptoms | Previous  treatment | Size  (mm) | Surgery | RT | Histology type | Ki-67 comparison  (%) | Postoperative  neurologic status and complications | Recurrence  (months) | Follow-up  (months) |
| --- | --- | --- | --- | --- | --- | --- | --- | --- | --- | --- | --- | --- |
| Case 1 | 53, F | No | Hearing loss, facial paralysis HB IV | 1. UR  2. GTR  3. GKS | 40 | NTR | No | MPNST | 2.0 vs. 3.5 vs. 30 | Hearing loss, FN function HB IV, aspiration & pneumonia | NA | D (6) |
| Case 2 | 67, F | No | Hearing loss, facial paralysis HB III | 1. NTR  2. GKS | 22 | 1. GTR  2. NTR | GKS | MPNST | 2.5 vs. 12.5 vs. 35 | Hearing loss, FN function HB III, pneumonia | R (31) | A (40) |
| Case 3 | 32, F | No | Decreased hearing for both sides, facial paralysis HB IV | 1. UR  2. GKS*2 | 60 | STR | No | MPNST | 1.5 vs. 6.5 | Decreased hearing for both side, FN function HB IV | NA | NA |
| Case 4 | 52, F | No | Facial paralysis HB III | None | 28 | GTR | No | MPNST | 22.5 | FN function  HB III | R (15) | D (2) |

A, alive; D, dead; FN, facial nerve; GKS, gamma knife radiosurgery; GTR, gross total resection; HB, House-brackmann; MPNST, malignant peripheral nerve sheath tumor; NA, not available; NF, neurofibromatosis; NTR, near total resection; Pt. No., patient number; R, recurrence; RT, radiotherapy; STR, subtotal resection; UR, unknown resection.
