# Supplementary material for: Malignant Transformation in Vestibular Schwannoma: Clinical Study With Survival Analysis
Source: Front Oncol. 2021 Apr 14;11:655260. doi: 10.3389/fonc.2021.655260 (PMC8079768; doi:10.3389/fonc.2021.655260)
Supplement: Supplementary file 1 [file DataSheet_1.zip › Supplementary Table 2.DOCX]

Supplementary Table 2: Pathological findings of our 4 MTVSs cases in consecutive surgeries

| Pt. No. | First operation | Second operation | Third operation |
| --- | --- | --- | --- |
| Case 1 | Benign VS; Ki-67 (+, 2.0%) | Benign VS; Ki-67 (+, 3.5%) | MPNST (WHO grade 3/FNCLCC grade 2); IHC positive: S-100*, nestin, Leu-7*, p53, p63, CgA, PCK, Oligo-2, synaptophysin, p40, CD99, Ki-67 (+, 30%); negative: GFAP, NF, PR |
| Case 2 | Benign VS; Ki-67 (+, 2.5%) | MPNST (WHO grade 3/FNCLCC grade 2); IHC positive: S100*, CR*, CD57*, IV collagen*, STAT-6, CD34, GFAP*, H3K27me3, P53; Ki-67 (+, 12.5%); negative: NF, EMA, Oligo-2 | MPNST (WHO grade 4/FNCLCC grade 3); IHC positive: S100*, CD34*, p53, H3K27me3, Ki-67 (+, 35%); negative: GFAP, H3K27m, desmin, myoD1, STAT |
| Case 3 | Benign VS; Ki-67 (+, 1.5%) | MPNST (FNCLCC grade 1); IHC positive: S-100*, Ki-67 (+, 6.5%); negative: EMA | - |
| Case 4 | MPNST (WHO grade 2/FNCLCC grade 1); positive: S100*, CR*, Ki-67 (+, 22.5%); negative: EMA, PR, STAT-6 | - | - |

FNCLCC, Fédération Nationale des Centres de Lutte Contre le Cancer; IHC, immunohistochemical staining; MPNST, malignant peripheral nerve sheath tumor; MTVS, malignant transformation of vestibular schwannoma; Pt. No., patient number.

* Indicate histopathological markers for vestibular schwannoma.
