# Supplementary material for: Malignant Transformation in Vestibular Schwannoma: Clinical Study With Survival Analysis
Source: Front Oncol. 2021 Apr 14;11:655260. doi: 10.3389/fonc.2021.655260 (PMC8079768; doi:10.3389/fonc.2021.655260)
Supplement: Supplementary file 1 [file DataSheet_1.zip › Supplementary Table 3.DOCX]

Supplementary Table 3: Summary of baseline characteristics in 71 patients with MTVSs

| Characteristic | Number or results |
| --- | --- |
| Age (years) |  |
| Median | 47.2 ± 17.5 |
| Range | 2-81 |
| Sex (n, %) |  |
| Male | 27 (40.9) |
| Female | 39 (59.1) |
| NF (%) | 12/65 (18.5) |
| Follow-up (months) |  |
| Median | 9.9 ± 9.5 |
| Range | 0-40 |
| Tumor size (mm) | 35.1 ± 13.2 |
| Pathology type |  |
| MPNST | 49 (69.0) |
| Others | 22 (31.0) |
| MIB-1 (%) |  |
| Median | 30.6 ± 18.0 |
| Range | 3.0-80.0 |
| Previous treatment (n, %) |  |
| Surgery | 8 (11.4) |
| RT | 16 (22.9) |
| Surgery & RT | 19 (27.1) |
| None | 27 (38.6) |
| Extent of resection (n, %) |  |
| GTR | 24 (36.4) |
| IR | 25 (37.9) |
| UR | 17 (25.8) |
| Radiotherapy (n, %) | 25/56 (44.6) |
| Progression (%) | 37/45 (82.2) |
| Death (%) | 47/64 (73.4) |

GTR, gross total resection; IR, incomplete resection; MTVS, malignant transformation of vestibular schwannoma; NF, neurofibromatosis; RT, radiotherapy; UR, unknown resection.
