# Supplementary material for: Malignant Transformation in Vestibular Schwannoma: Clinical Study With Survival Analysis
Source: Front Oncol. 2021 Apr 14;11:655260. doi: 10.3389/fonc.2021.655260 (PMC8079768; doi:10.3389/fonc.2021.655260)
Supplement: Supplementary file 1 [file DataSheet_1.zip › Supplementary Table 4.DOCX]

Supplementary Table 4: Univariate analysis of prognostic factors for OS

|  |  | OS | | |
| --- | --- | --- | --- | --- |
| Factor | Categories | Numbers | Chi-square | Log-rank P value |
| Age | Age ≤ 46y  Age > 46y | 22  34 | 0.542 | 0.462 |
| Sex | Male  Female | 24  32 | 0.370 | 0.543 |
| NF disorder | Yes  No | 10  45 | 2.673 | 0.102 |
| Size | Size ≤ 30mm  Size > 30mm | 21  24 | 3.934 | **0.047** |
| MIB-1 | MIB-1 ≤ 30%  MIB-1 > 30% | 17  13 | 0.312 | 0.576 |
| Radiation history | Yes  No | 28  29 | 0.248 | 0.618 |
| Surgical history | Yes | 23 | 0.036 | 0.849 |
|  | No | 36 |  |  |
| Radiation or surgical history | Yes | 35 | 1.204 | 0.273 |
|  | No | 24 |  |  |
| Extent of resection | GTR  IR | 20  25 | 1.152 | 0.283 |
| Adjuvant radiotherapy | Yes  No | 25  26 | 10.961 | **0.001** |
| Pathologic type | MPNST  Others | 42  17 | 0.840 | 0.359 |

GTR, gross total resection; IR, incomplete resection; MPNST, malignant peripheral nerve sheath tumor; NF, neurofibromatosis; OS, overall survival.
