# Supplementary material for: Malignant Transformation in Vestibular Schwannoma: Clinical Study With Survival Analysis
Source: Front Oncol. 2021 Apr 14;11:655260. doi: 10.3389/fonc.2021.655260 (PMC8079768; doi:10.3389/fonc.2021.655260)
Supplement: Supplementary file 1 [file DataSheet_1.zip › Supplementary Table 5.DOCX]

Supplementary Table 5: Multivariate analysis of prognostic for OS

| Prognostic factor | P value | HR | 95% CI |  |
| --- | --- | --- | --- | --- |
|  |  |  | Lower | Upper |
| Size | 0.092 | 1.899 | 0.901 | 4.001 |
| Adjuvant radiotherapy (yes/no) | **0.005** | 0.359 | 0.175 | 0.737 |

CI, confidence interval; HR, hazard ratio; OS, overall survival.
