# Supplementary material for: Malignant Transformation in Vestibular Schwannoma: Clinical Study With Survival Analysis
Source: Front Oncol. 2021 Apr 14;11:655260. doi: 10.3389/fonc.2021.655260 (PMC8079768; doi:10.3389/fonc.2021.655260)
Supplement: Supplementary file 1 [file DataSheet_1.zip › Supplementary Table 6.DOCX]

Supplementary Table 6: Key characteristics of malignant transformation of vestibular schwannomas totally conform modified Cahan’s criteria [7, 8]

| Pt. No. | Investigator | Age (years),  Sex | NF | Previous Treatment | Latency (months) | Size(mm) | Surgery | RT | Histology Type | Ki-67(%) | R/ME | Follow-Up  (months) |
| --- | --- | --- | --- | --- | --- | --- | --- | --- | --- | --- | --- | --- |
| 1 | Thomsen et al. (45), 2000 | 25, F | NF2 | SRS | 72 | NA | UR | GKS | sarcoma | NA | NA | D (0.2) |
| 2 | Hanabusa et al. (4), 2001 | 51, F | no | GTR& GKS | 15 | 21 | 1. STR  2. UR  3. UR | GKS | sarcoma | NA | R | D (14) |
| 3 | Shin et al. (27), 2002 | 32, F | no | PTR&GKS | 72 | 35 | UR&CT | none | MPNST | NA | R/ME | D (10) |
| 4 | Wilkinson et al. (46), 2004 | 53, M | no | UR&RT | 48 | NA | UR | none | MPNST | 20 | NA | D (8) |
| 5 | Kubo et al. (47), 2004 | 53, M | no | 1. STR  2. GTR  3. GKS&CT | 12 | 30 | 1. GTR  2. UR | RT | MPNST | 55.9 | R | D (11) |
| 6 | Maire et al. (48), 2006 | 45, F | no | PR&FRT | 216 | 35 | GTR | none | MPNST | 15 | NA | NA |
| 7 | Akamatsu et al. (49), 2010 | 81, F | no | 1. STR  2. PR&GKS | 96 | 45 | GTR | FRT | MPNST | 40 | NA | NA |
| 8 | Carlson et al. (43), 2010 | 26, F | NF2 | 1. UR  2. STR& SRS  3. UR | 120 | 30 | IR | none | rhabdomyosarcoma | high | NA | D (3) |
| 9 | Demetriades et al. (50), 2010 | 37, M | no | 1. PR&GKS  2. UR | 120 | 45 | 1. GTR  2. UR | none | MPNST | NA | R | D (4) |
| 10 | Yang et al. (51), 2010 | 74, M | no | UR&SRS | 72 | 25 | GTR | none | sarcoma | NA | NA | D (1) |
| 11 | Markou et al. (52), 2012/Maire et al. (48), 2006† | 61, F | no | 1. PR  2. FRT | 5 | 35 | GTR | none | MPNST | 15 | R | A (12) |
| 12 | Newell & Pollack (53), 2012 | 50, M | no | yes | NA | NA | UR | NA | MPNST | NA | ME | D (3) |
| 13 | Puataweepong et al. (54), 2012 | 41, F | no | PR*2&SRS | 72 | 45 | PR | RT | MPNST | 20 | R | D (2) |
| 14 | Yanamadala et al. (28), 2013 | 51, F | no | 1. STR&GKS  2. STR | 59 | 36 | 1. GTR  2. UR  3. UR | CT | MPNST | 33.8 | R | D (11) |
| 15 | Seferis et al. (8), 2014 | 34, F | no | STR&GKS | 72 | 8.4cc | PR | GKS | MPNST | 30 | No | A (~18) |
| 16 | Wolf et al. (55), 2018 | NA, NA | NA | UR&SRS | 104 | NA | UR | NA | MPNST | NA | NA | D (~24) |
| 17 | Peker et al. (56), 2018 | 40, F | no | GTR&SRS | NA | 40mm | 1. GTR  2. STR | none | MPNST | 6 | R | NA |
| 18 | Present study | 53, F | no | 1. UR  2. GTR  3. GKS | 12 | 40mm | GTR | none | MPNST | 30 | NA | D (6) |
| 19 | Present study | 67, F | no | 1. NTR  2. GKS | 26 | 22mm | 1. GTR  2. NTR | GKS | MPNST | 12.5 | R | A (40) |
| 20 | Present study | 32, F | no | 1. UR  2. GKS*2 | 20 | 60mm | STR | no | MPNST | 6.5 | NA | NA |

† These two authors described the same patient.

A, alive; cc, cubic centimeter; CT, chemotherapy; D, dead; F, female; FRT, fractionated radiotherapy; GKS, gamma-knife radiosurgery; GTR, gross total resection; IR, incomplete resection; M, male; ME, metastasis; MPNST, malignant peripheral nerve sheath tumor; NA, not available; NF, neurofibromatosis; NTR, near total resection; PR, partial resection; R, recurrence; RT, radiation therapy; SRS, stereotactic radiosurgery; STR, subtotal resection; UR, unknown resection.
